# The Effectiveness of Psychosocial Interventions for Elder Abuse in Community Settings: A Systematic Review and Meta-Analysis

**DOI:** 10.3389/fpsyg.2021.679541

**Published:** 2021-05-26

**Authors:** Yan Shen, Fei Sun, Anao Zhang, Kaipeng Wang

**Affiliations:** ^1^School of Economic & Management and Law, Hubei Normal University, Huangshi, China; ^2^School of Social Work, Michigan State University, East Lansing, MI, United States; ^3^School of Social Work, University of Michigan, Ann Arbor, MI, United States; ^4^Graduate School of Social Work, University of Denver, Denver, CO, United States

**Keywords:** elder abuse, psychosocial intervention, community settings, systematic review, meta-analysis

## Abstract

As a global public health concern, elder abuse negatively affects health, psychosocial wellbeing, and mortality among elders. Research and practice efforts made to explore effective prevention and intervention strategies are growing. Despite the growing number of intervention studies on elder abuse, research synthesis on the empirical literature seems lacking. This study aims to identify the pooled effect size of prevention and interventions targeted ultimate and intermediate outcomes for elder abuse that occurred in community settings. Following the Cochrane guideline, our team searched across eight electronic databases and manually searched reference lists of eligible studies and existing systematic reviews for all potentially eligible studies. A random-effects model of 51 effect size estimates reported an overall positive and statistically significant treatment effect of psychosocial interventions for elder abuse, *d* = 0.63, *p* < 0.05. The overall treatment effect was approaching statistical significance at 0.1 level for ultimate outcomes, *d* = 0.32, *p* = 0.09, and intermediate outcomes, *d* = 0.75, *p* = 0.1. An overall significant effect size was found among family-based interventions, *d* = 0.59, *p* < 0.05, and interventions targeting older adults and their caregivers, *d* = 0.45, *p* < 0.05. Existing evidence supports an overall significant effect for psychosocial interventions for elder abuse. Interventions that used a family-based model, combined education and supportive services, and targeted both caregivers and elders, showed significant effect size, suggesting such features being considered in elder abuse intervention design. Future intervention research is needed to shed light on the link between intervention activities and ultimate change in elder abuse behaviors.

## Introduction

Elder abuse refers to intentional or unintentional harmful acts toward an older person where trust is expected. Common types of elder abuse include physical, psychological, sexual abuse, financial exploitation, and neglect (World Health Organization, 2002; Acierno et al., 2010). As a global public health concern, about 1 in 6 community-dwelling older adults experienced some form of abuse in the last 12 months as found in a meta-analysis that explored prevalence rates of elder abuse (Yon et al., 2017). The prevalence rate of abuse in elder care facilities is even higher as two-thirds of care staff members reported abusive behaviors (Yon et al., 2019). The profound impact of elder abuse on victim’s health, finances, quality of life, and even mortality (Acierno et al., 2010) deserves attention.

Current elder abuse interventions include public education and advocacy, caregiver support, psychological support for victims, care coordination, and multidisciplinary case management to name a few (Acierno et al., 2010). Based on existing literature, programs that suggest the effectiveness of elder abuse are through providing (1) caregiver supportive services (Livingston et al., 2013), (2) money management coaching (Sacks et al., 2012), (3) telephone helplines (van Bavel et al., 2010), (4) emergency shelters for older victims (Heck and Gillespie, 2013), and (5) access to a multidisciplinary team (Teaster et al., 2003). Educational or training interventions are more accessible than supportive services or case management interventions (Sacks et al., 2012; Livingston et al., 2013). Elder abuse preventions and interventions tend to achieve two types of outcomes: reducing the occurrence of abusive behaviors (Hsieh et al., 2009; Khanlary et al., 2016), and mitigating elder abuse risk factors, such as psychosocial stress and lack of awareness or competency among nursing staff, family caregivers, and older adults themselves (Pellfolk et al., 2010; Baker et al., 2016; Estebsari et al., 2018).

Most meta-analysis studies regarding elder abuse focus on the prevalence and risk factors rather than on elder abuse intervention effects (Yon et al., 2017, 2019). Despite the growing number of intervention studies on elder abuse, research synthesis on the pooled effect for elder abuse interventions remains lacking. From the limited available systematic reviews of elder abuse intervention programs, either found a lack of sufficient evidence to establish the effectiveness of elder abuse (Ploeg et al., 2009; Alt et al., 2011; Fearing et al., 2017) programs or programs that did not use rigorous evaluations to assess the effectiveness of said programs (Rosen et al., 2019).

Furthermore, the only meta-analysis to our knowledge synthesized 24 studies of elder abuse interventions (Ayalon et al., 2016). This study found an overall small but significant treatment effect for restraint use reduction, *d* = −0.24, 95% CI (−0.38, −0.09). While highly valuable, this meta-analysis primarily focused on the restraint use as an abuse outcome, which can be questionable as the use of physical restraints with a physician’s order can be medically necessary rather than abusive.

Despite the above evidence that supports the potential effect of intervention programs for elder abuse, the conclusion is far from definitive because of the limited number of studies and a narrowed scope of outcomes reviewed in these systematic reviews (Ploeg et al., 2009; Alt et al., 2011; Fearing et al., 2017). Elder abuse preventions and interventions typically aim for changes in intermediate or ultimate outcomes. Ultimate outcomes refer to the reduction in the occurrence or reoccurrence of abusive behaviors, while intermediate outcomes include the mitigation of risk factors (e.g., psychosocial stress) and promotion of protective factors (e.g., improving knowledge of abuse and enhancing the competency of addressing abuse) that will lead to the reduction of elder abuse. Both intermediate and ultimate outcomes need to be considered for a competent narrative for the effectiveness of elder abuse interventions.

Therefore, our systematic review and meta-analysis study aims to examine prevention and intervention studies that targeted ultimate and intermediate outcomes for elder abuse that occurred in community settings. Acknowledging that these two outcome types of elder abuse, though interrelated, reflect distinctive features, we hence examined the pooled effect of all outcomes combined and the respective effect for different outcome types.

## Materials and Methods

### Search Strategy

The identification of relevant studies was performed in two steps. The first step consisted of searching eight academic databases from Jan 1990 to December 2020: Cumulative Index to Nursing and Allied Health (CINAHL), Cochrane Library/Central Register of Controlled Trials, Database of Abstracts of Reviews of Effects (DARE), PsycINFO, PubMed, MEDLINE, BIOSIS, and Science Direct. Key search terms included “older abuse,” “elder abuse,” “elder neglect,” “elder mistreatment/maltreatment,” or “older neglect” and “physical abuse,” or “emotional abuse” or “financial abuse” and “intervention” or “program.” To identify the right design of the intervention, search terms included “control group” or “RCT.” The set of keywords was used for both study title and study abstract search across databases. Second, the reference lists of studies and systematic reviews identified from the database search were reviewed for additional relevant studies.

### Inclusion Criteria

The inclusion criteria for studies published in English are those that (1) assessed the effectiveness of a prevention or intervention program designed to address abuse or neglect of elders aged 65 or older living the community settings, (2) targeted at elders or family members, (3) used randomized controlled trials (RCTs) or controlled trials (without random assignment), (4) reported at least one elder abuse intervention outcome, and (5) reported statistical information sufficient to calculate effect size required for meta-analysis. We did not limit the location of the studies. Outcomes consisted of (1) ultimate outcomes: occurrence or reoccurrence of elder abuse behaviors; and (2) intermediate outcomes: reduction of risk factors for elder abuse, such as reduced stress, improved knowledge of abuse, and enhanced competency of addressing abuse.

### Data Collection and Extraction

Three researchers conducted data collection and extraction. Two reviewers (YS and FS) screened titles and abstracts for eligible studies independently with decisions blind to one another. If any disagreement existed, two reviewers would discuss first and if unresolvable, a third reviewer (AZ) would intervene and make a final decision. The three-person team adopted the same process in later full article review and quality assessment. Inter-screener reliability was 92% and inter-rater reliability of full articles was 85%.

Measures of ultimate outcomes included occurrence or reoccurrence of elder abuse, here termed as behavioral outcomes, and intermediate outcomes (i.e., risk or protective factors for elder abuse) included psychosocial stress, knowledge of elder abuse, and competency of addressing elder abuse.

Basic information extracted from studies consisted of participant demographic characteristics (i.e., age, gender, and ethnicity), geographic areas of the study (i.e., United States, Asia, and Europe), and study design (i.e., RCT and non-randomized controlled trial). Intervention characteristics consisted of intervention type (i.e., family, individual, and mixed), target population (i.e., elders, family caregivers, or both), intervention approach (i.e., education, supportive services, and mixed), and intervention frequency and duration.

### Risk of Biases Assessment

Quality of studies was assessed using the Jadad scale (Halpern and Douglas, 2005), also known as the Oxford quality scoring system. Studies’ risk of bias was assessed using the Cochrane Collaboration’s tool for risk of bias in randomized trials (Alderson et al., 2008). The research team resolved discrepancies and reached a consensus on these ratings. Publication bias was assessed using a funnel plot (visual analysis) and the Vevea and Wood sensitivity weighted function analysis (statistical analysis; Vevea and Woods, 2005). For funnel plot, we plotted independent effect sizes only first and then plotted all effect sizes with some of them are dependent of each other.

### Meta-Analytic Procedures

We used the R software for data analysis. Treatment effect sizes were estimated for each individual study to determine treatment clinical effects. For continuous outcomes, the standardized mean difference (SMD) was calculated to obtain Hedges’ g statistic (Cooper et al., 2009). For binary outcomes, an odds ratio (OR) was calculated first, followed by taking the log transformation of the odds ratio (i.e., log odds ratio). The log OR statistic was further transformed into the same effect size metric as the Hedges’ g statistic using procedures suggested by Cooper et al. (2009). The Hedges’ g was further bias corrected using a J function (Cooper et al., 2009) to obtain an unbiased estimation of the treatment effect, noted as *d* for the rest of the text. When meta-analyzing the effect size estimates, we used the inverse variance weight, which is considered as an optimal weight estimate in meta-analysis (Marín-Martínez and Sánchez-Meca, 2010).

Between-study and between-effect size, heterogeneity was assessed using multilevel modeling with R’s *metafor* package (Viechtbauer, 2010). A pooled overall treatment effect and potential moderator analyses were achieved through meta-regression with robust variance estimation (RVE) using R’s *robumeta* package (Tanner-Smith et al., 2016). The intercept only in the meta-regression model offered overall averages of treatment effect sizes across studies; and models with covariates allowed the identification of effects of potential moderators on treatment effect sizes. Meta-regression using the RVE method effectively handles the statistical dependence created by one study reporting multiple effect size estimates on the same outcome (Hedges et al., 2010). For example, a study may use more than one measure to evaluate a provider’s knowledge of elder abuse, resulting in the two knowledge measures within the same study being potentially dependent on each other (see Table 1, outcome measures). The RVE approach not only effectively addresses the dependent issues but also produces robust estimation regardless of the heterogeneity assumption, meaning results robust across fixed- and random-effects models (Hedges et al., 2010). Given the small number of studies included in this review, we also conducted a small sample size correction to the meta-regression analysis, and for an estimate with degrees of freedom greater than 4, *p* < 0.05 is considered statistically significant. For an estimate with degrees of freedom lower than 4, *p* < 0.01 is considered statistically significant. Sensitivity analyses were conducted by averaging dependent effect sizes within each study. Because both methods produced the same statistical inference, we reported the RVE results in this paper (Tipton, 2015).

**Table 1:** Study characteristics of six included studies.

Author	Geo.	Setting	Design	Demographics	Intervention target	Sample size (baseline)	Intervention type and approach	Intervention duration	Outcome measures
Filinson (1993)^*^	United States	Community based	CT	Older adults 73.4 years old 81% female	Older adults and their families	Eighty-four elders and their caregivers T = 42; C = 42	Elderly Abuse Support Project	Eightteen-month period	Social isolation; accessing service; legal action
Davis et al. (2001)	United States	Community based	RCT	Older adults 65 years old (median) 81% female	Older adults	Four hundred and three elders T = 202, C = 201	Public education and home visit	six months follow-up 12 months follow-up	CTS
Rabiei et al. (2013)	Iran	Community based	CT	Older adults 67.9 years old 51.6% female	Older adults and their families	Sixty-four elders and their families T = 32, C = 32	Family-based empowerment education	Ten forty-five-min sessions, 3-month period	Self-efficacy; self-esteem; perceived threat
Khanlary et al. (2016)	Iran	Community based	RCT	Older adults 65 years old 14.8% female	Older adults and their families	Thirty elders and their families T = 15, C = 15	FBCBSW	Five forty-five-min sessions	DEAQ
Cooper et al. (2016)^*^	UK	Community based	RCT	Caregivers: 58.2 years old 68.5% female Older adults: 79.0 years old 58.5% female	Family caregivers	Two hundred and sixty caregivers T = 173; C = 87	A psychological intervention START	Eight-session, short term (4 and 8 month) and long term (12 and 24 months)	MCTS
Estebsari et al. (2018)^*^	Tehran.	Community based	RCT	Older adults 65.9 years old 50% female	Older adults	Four hundred and sixty-four older adults T = 232, C = 232	An empowerment educational intervention to prevent elder abuse.	Twenty 45-to-60-min training sessions over 6-month, 18-month period	Elder abuse knowledge questionnaire; SCARED questionnaire

CTS, Conflict Tactics Scale; FBCBSW, Family-Based Cognitive Behavioral Social Work; DEAQ, Domestic Elder Abuse Questionnaire; START, STrAtegies for RelaTives; MCTS, Modified Conflict Tactic Scale; SCARED, Stress level, Coping, Argument, Resources, Events, and Dependence.

* Indicates effect sizes were estimated through transformation of odds ratio.

## Results

### Search Results

**Figure 1:**
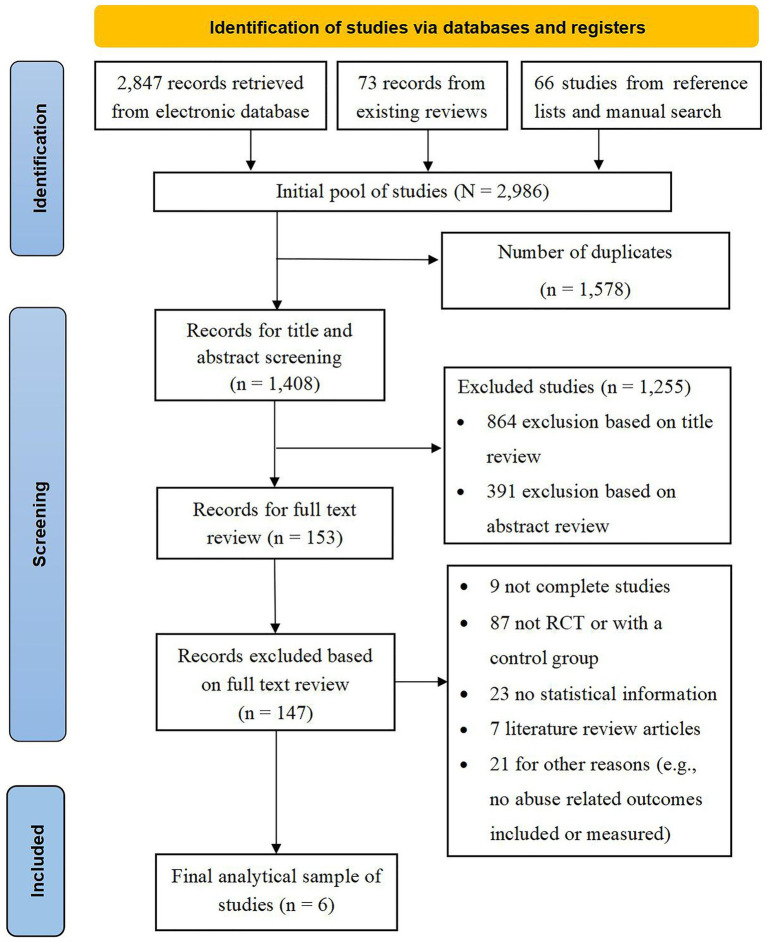
PRISMA chart of literature search.

The review process is summarized in Figure 1. A total of 2,986 studies were identified through a comprehensive search strategy for interventions to prevent or stop elder abuse in the community or an institutional setting. After 1,578 duplicate studies were removed, 1,255 studies were further excluded based on a title and abstract review. Of the remaining 153 studies, 147 studies were excluded for reasons, such as single arm trial without a control group or without statistical data, resulting in an analytical sample of six studies, containing 51 effect size estimates, in the final meta-analysis.

### Characteristics of Included Studies

Study characteristics are presented in Table 1. Of the six primary studies, four studies (67%) were published after 2010 and one (17%) was published in the 1990s. The six studies included a total sample of 1,305 participants. Participants’ ages averaged at 64.65 (*SD* = 4.15), and 64.52% were female (*SD* = 17.13). Four studies (66.7%) included at least one intermediate outcome measure and four studies (66.7%) included at least one ultimate outcome on elder abuse. Specifically, three studies included psychosocial stress outcomes, four studies encompassed outcomes related to knowledge and competency, and four studies addressed ultimate abuse-related behavioral outcomes.

Most interventions in reviewed studies were delivered in a family format (*n* = 3, 50%), which lasted on average over 10 months (*SD* = 7.55, range = 3–18 months). Two individual-based interventions (33.3%) were on average 21 months in duration (*SD* = 4.24, range = 18–24 months) across studies. Three studies directed at both older adults and family members, two studies targeted older adults and one study targeted family members. Two interventions adopted an education approach, three used supportive services, and one incorporated both educational and supportive service interventions. Study sites covered Europe (*n* = 2, 33.3%), the United States (*n* = 2, 33.3%), and Asia (*n* = 2, 33.3%).

### Quality Assessment of Included Studies and Risk of Bias

Both RCT studies (*n* = 4) and non-randomized controlled trial studies (*n* = 2) were rated using the Jadad scale (Halpern and Douglas, 2005) for reporting controlled trials and the Cochrane risk of bias assessment tool. Using the Jadad scale (see Supplementary Table 1), the six trials had an average score of 2.8 (*SD* = 1.17) out of 5, indicating acceptable quality. These studies were rated satisfactory in mentioning randomization (5/6), tracking all participants (6/6), and randomization (4/6). However, these studies done were not satisfactory using appropriate blinding, just two studies mentioned blinding. Using the Cochrane Collaboration’s tool for assessing the risk of bias (see Supplementary Table 2), studies were rated most satisfactorily in selective outcome reporting (6/6), random sequence generation (4/6), and handling incomplete outcome data (4/6). Risk of bias was observed in allocation concealment (0/6), blinding of study participants and personnel (0/6), and blinding of outcome data assessment (2/6).

**Figure 2:**
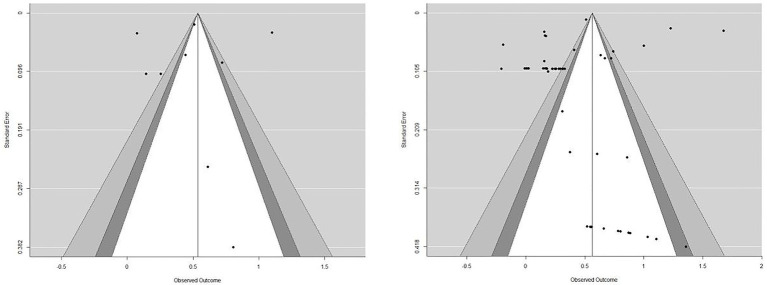
Funnel plot for publication bias. We plotted independent effect sizes (*n* = 9 from 6 studies) in the left plot and plotted all effect sizes (some dependent of each other) in the right plot.

Both visual and statistical examination suggested an absence of publication bias (see Figure 2). The funnel plot seemed reasonably symmetric and showed no concerning outliers. The Vevea and Wood sensitivity weighted function analysis further confirmed the absence of publication bias.

### Meta-Analysis Results and Subgroup Analyses

Multilevel modeling and the *Q* statistic used in heterogeneity assessment indicated a significant amount of heterogeneity across both effect size estimates and studies [*Q*(50) = 5914.3, *p* < 0.01], suggesting a random-effects model is appropriate. We found an overall positive and statistically significant treatment effect (*d* = 0.63, 95% CI [0.02, 1.24], *p* < 0.05).

Effect sizes were found depending on outcomes in subgroup analyses. The overall treatment effect was approaching statistical significance at 0.1 level for ultimate outcomes (*d* = 0.32, 95% CI [−0.1, 0.73], *p* = 0.09) and intermediate outcomes (*d* = 0.75, 95% CI [−0.27, 1.77], *p* = 0.10). More specifically for intermediate outcomes, effect size for psychosocial stress outcomes was 0.57 (95% CI [−0.29, 1.44], *p* = 0.1), and for knowledge and competency outcomes (*d* = 0.66, 95% CI [−0.42, 1.75], *p* = 0.15).

In terms of intervention characteristics, we found that significant effect sizes across intervention type and target population. An overall significant effect size was found among family-based interventions (*d* = 0.59, 95% CI [0.18, 1.01], *p* < 0.05), but not among individual-based interventions (*d* = 0.86, 95% CI [−9.08, 1.8], *p* = 0.47). In terms of targeted populations, an overall statistically significant treatment effect was observed among interventions targeting both entities (e.g., older adults and their family caregivers; *d* = 0.45, 95% CI [0.01, 0.89], *p* < 0.05), but not among those targeting sole older adults or family caregivers.

Subgroup analyses using other study characteristics failed to show significance. Both RCT and non-randomized controlled trial studies reported non statistically significant treatment effect size (*d* = 0.67, 95% CI [−0.51, 1.85], *p* = 0.17) and (*d* = 0.54, 95% CI [−0.3, 1.38], *p* = 0.08) respectively. Subgroup analyses among studies published in the United States, Europe, and Asia did not yield any significance either. Specific results are presented in Table 2.

**Table 2:** Overall and sub-group meta-analysis.^†^

	Estimate	*t* (*df*)	K/N	95% CI	*p*
Overall treatment effect	0.63	2.64 (4.99)	6/51	[0.02, 1.24]	*p* < 0.05
**Outcome type I**					
Intermediate outcomes	0.75	2.33(3)	4/25	[−0.27, 1.77]	*p* = 0.10
Ultimate outcomes	0.32	2.59 (2.69)	4/26	[−0.1, 0.73]	*p* = 0.09
**Outcome type II**					
Psychosocial stress	0.57	2.99(1.91)	3/8	[−0.29, 1.44]	*p* = 0.10
Knowledge and competency	0.66	1.94(3)	4/17	[−0.42, 1.75]	*p* = 0.15
Behavior outcomes	0.32	2.59 (2.69)	4/26	[−0.1, 0.73]	*p* = 0.09
**Intervention type**					
Family	0.59	6.68(1.84)	3/23	[0.18, 1.01]	*p* < 0.05
Individual	0.86	1.09 (1)	2/9	[−9.08, 1.8]	*p* = 0.47
Mixed	--	--	1/19	--	--
**Target population**					
Mixed population	0.45	3.52(2.65)	4/42	[0.01, 0.89]	*p* < 0.05
Older adults^††^	--	--	1/5	--	--
Caregivers^††^	--	--	1/4	--	--
**Intervention approach**					
Education	1.13	2.2 (1)	2/8	[−5.39, 7.65]	*p* = 0.27
Support	0.39	1.97 (1.85)	3/24	[−0.53, 1.30]	*p* = 0.20
Mixed approaches	--	--	1/19	--	--
**Intervention design**					
Randomized controlled trials	0.67	1.8 (2.99)	4/40	[−0.51,1.85]	*p* = 0.17
Non-randomized controlled trials	0.54	8.11 (1)	2/11	[−0.3, 1.38]	*p* = 0.08
**Study location**					
United States	0.33	2.14 (1)	2/27	[−1.61, 2.26]	*p* = 0.28
Europe	0.86	1.09(1)	2/9	[−9.08, 1.8]	*p* = 0.47
Asia	0.67	7.53 (1)	2/15	[−0.46, 1.8]	*p* = 0.08

K, number of studies; N, number of effect size estimates.

† If df < 4, coefficient significance should be interpreted with caution.

†† Coefficient cannot be estimated because only 1 study falls under this category.

### Moderator Analyses

Results of moderator analyses are presented in Table 3. Only two univariate meta-regression models revealed a significant moderator that explains the heterogeneity between studies and effect sizes. Interventions targeting older adults reported significantly greater treatment effects than those targeting caregivers, or both. Intervention using a family centered approach had a greater treatment effect than the intervention delivered using a mixed format which included group and individual interventions.

**Table 3:** Single-predictor meta-regression analysis.

	Estimate	*t* (*df*)	K/N	95% CI	*p*
Outcome type I (*ref: intermediate outcomes*) *b*_0_	0.83	2.07 (2.46)	6/51	[−0.62, 2.28]	*p* = 0.15
Ultimate *b*_1_	−0.42	−0.94 (4.40)	6/51	[−1.62, 0.78]	*p* = 0.39
Outcome type (*ref: psychosocial stress*) *b*_0_	0.58	2.37 (1.68)	6/51	[−0.68, 1.83]	*p* = 0.17
Knowledge and competency *b*_1_	0.34	0.65 (2.16)	6/51	[−1.79, 2.48]	*p* = 0.58
Behavior *b*_2_	−0.17	−0.53 (2.41)	6/51	[−1.31, 0.98]	*p* = 0.64
Intervention type (*ref:Family*) *b*_0_	0.63	6.67 (2.00)	6/51	[−0.22, 1.03]	*p* = 0.02
Individual *b*_1_	−0.23	0.29 (2.32)	6/51	[−2.75, 3.21]	*p* = 0.80
Mixed *b*_2_	−0.45	−4.83 (2.00)	6/51	[−0.86, −0.05]	*p* = 0.04^*^
Target population (*ref:older adults*) *b*_0_	1.64	2e + 16 (3.79)	6/51	[1.64, 1.64]	*p* = 0.00
Caregiver *b*_1_	−1.56	inf (4.53)	6/51	[−1.56, −1.56]	*p* = 0.00^*^
Mixed *b*_2_	−1.13	−8.54 (2.98)	6/51	[−1.55, −0.71]	*p* = 0.00^*^
Intervention approach (*ref: education*) *b*_0_	1.14	2.22 (1.00)	6/51	[−5.38, 7.65]	*p* = 0.27
Support *b*_1_	−0.70	−1.27 (2.29)	6/51	[−2.82, 1.42]	*p* = 0.32
Mixed *b*_2_	−0.97	−1.88 (1.00)	6/51	[−7.48, 5.55]	*p* = 0.31
Type of design (*ref: RCT*) *b*_0_	0.67	1.80 (2.99)	6/51	[−0.52, 1.85]	*p* = 0.17
Non-RCT *b*_2_	−0.13	−0.33 (2.08)	6/51	[−1.69, 1.44]	*p* = 0.77
Study location (*ref: United States*) *b*_0_	0.33	2.13 (1)	6/51	[−1.61, 2.26]	*p* = 0.28
Europe *b*_1_	0.53	0.67 (2)	6/51	[−2.90, 3.96]	*p* = 0.57
Asia *b*_2_	0.38	2.10 (2)	6/51	[−0.40, 1.16]	*p* = 0.17

K, number of studies; N, number of effect sizes.

* If *df* < 4, coefficient significance should be interpreted with caution. Although presented in the same table, predictors were entered one-by-one individually to the model. “inf” denotes the number is infinitely large.

## Discussion

Our major finding identified a statistically significant effect size (*d* = 0.63) of psychosocial interventions for elder abuse, suggesting that the effectiveness of available elder abuse prevention and interventions are moderately supported by evidence. Addressing elder abuse needs a multisystem effort that targets various change agents (e.g., elders, family, and caregivers) and along with multiple domains such as abuse awareness, knowledge, and behaviors (World Health Organization, 2002). Although the overall effect size sounds promising, the findings from the subgroup analyses shed further light on the effectiveness regarding different intervention outcomes and provided specific directions for future research.

The treatment effect for intermediate outcomes (*d* = 0.75, *p* = 0.1) and for the ultimate outcomes (*d* = 0.19, *p* = 0.09) approached significance at 0.1 level. Because of the small power due to a limited number of articles included in the meta-analysis, these findings, though should be interpreted with caution, appear encouraging and promising. Intermediate outcomes can be viewed as mediators which bring about elder abuse reduction. Interventions reviewed in this study leaned toward an effective impact on intermediate outcomes, such as psychosocial stress, knowledge, and competency. This finding corresponds to a conclusion based upon an earlier Cochrane review of interventions for abuse prevention (Baker et al., 2016). As abuse-related knowledge outcomes are often treated as intermediate outcomes, research needs to improve their knowledge-related outcome measures to capture notable changes in abuse awareness and attitude, along with their impact on the occurrence of elder abuse. Moreover, research should not stop arriving at satisfactory intermediate outcomes, but rather continue to disclose the pathway between changes in psychosocial stress and abuse reduction.

Effect size is significant among interventions that were family-based, geared toward elders, and family members, and adopted multiple approaches (e.g., combining education and services). The effectiveness of family-based interventions suggests the change of family dynamics can mitigate abuse. Consistently, an overall statistically significant treatment effect was observed among interventions targeting both elders and family members. As we had a limited number of interventions solely on elders or caregivers, we could not speculate the effect size for each respective group. Elder abuse mostly occurs in family settings, where perpetrators are most family members. Thus, targeting family issues through mitigating risk factors on both sides, potential victims and perpetrators, are likely to yield ideal outcomes (Dong, 2015).

The use of various intervention approaches (e.g., education and supportive services) appeared to be effective, as mixed approach interventions showed an overall significant treatment effect. As there are multiple risks for elder abuse, intervention using mixed approaches can be more effective than interventions using one approach. Future interventions need to incorporate education, support, and services to assist older victims. Enhancing one’s awareness of elder abuse through education is important to the prevention of abuse; however, information itself is far from sufficient to trigger changes in attitude and behavior. Often, elders vulnerable to abuse or have been abused are likely to have other pressing social, health, financial, and legal needs. Similarly, mixed approaches better serve family caregivers who need tangible support to help manage challenging care tasks and emotional support for stress and burden.

Several limitations are inherent to systematic review and meta-analysis studies that should be noted. First, chances could be that one or more studies were missed in our search.

The total number of studies included was small, and a limited sample in subgroup analyses may have failed to show significance due to insufficient power. The small sample size, i.e., a small number of included studies, also prevented us from conducting certain subgroup analyses such as an overall treatment effect for interventions targeting different target population. Future studies should consider these analyses when more studies become available. Second, while two coders independently coded all studies and a team of experienced researchers resolved any disagreements, the results of this study are still subject to human errors. Third, as we used an advanced method to synthesize effect size estimates across outcomes, individual outcome contains distinctiveness in conceptualization. In the subgroup outcome analyses, some outcomes may suffer from a small sample size. As a result, we were unable to identify if the non-significant findings were due to low power. The same issue may exist in the moderator analyses, which prevented us from drawing any definitive conclusions. Fourth, as we focused on our search for psychological interventions, we did not include legal or policy interventions. As this area may play a role in elder abuse, these should be considered in future studies. Furthermore, research with a larger number of studies included will help explain the variations across effect sizes and studies.

## Conclusion

This study represents an initial effort to examine the pooled effect of elder abuse preventions and interventions *via* meta-analyses. Existing evidence is supportive of a modest effect (approaching significance at 0.1 level) of psychosocial interventions for elder abuse. Evidence appears promising for interventions on modifiable intermediate outcomes such as psychosocial stress, knowledge, and competency that are typically theorized to lead to changes in elder abuse occurrence, as well as interventions targeting ultimate outcomes (i.e., abuse reduction). Interventions that used a family-based model, combined education and supportive services, and targeted both caregivers and elders, showed significant effect size, suggesting such features incorporated in elder abuse intervention design. Yet, more research evidence is still needed, in particular, research that will shed further light on the link between intervention activities and changes in elder abuse behaviors.

Community-based approach that draws on the concerted efforts from multiple stakeholders (e.g., elders, families, and service professionals) and tackles multi-domain elder abuse risk factors (e.g., knowledge, competency, and support) is worth pursuing (Dong, 2015). Geriatric health and social care providers as well public health workers in the field of aging should be updated on the status of currently available interventions, adapt evidenced-informed interventions, and account for the heterogeneity of factors at the individual, agency, and cultural levels when promoting a safe and free of abuse environment for older adults.

## Data Availability Statement

The raw data supporting the conclusions of this article will be made available by the authors, without undue reservation.

## Author Contributions

YS screened and coded all studies, drafted the entire manuscript. FS screened and coded all studies, drafted the discussion section of the manuscript, and proofread the entire manuscript. AZ designed the search strategy, resolved conflict, conducted preliminary analysis, drafted the entire manuscript, and proofread the entire manuscript. KW conducted all the meta-analysis and led the writing of the method and result section and proofread the entire manuscript. All authors contributed to the article and approved the submitted version.

### Conflict of Interest

The authors declare that the research was conducted in the absence of any commercial or financial relationships that could be construed as a potential conflict of interest.

## References

[ref1] Acierno R. Hernandez M. A. Amstadter A. B. Resnick H. S. Steve K. Muzzy W.. (2010). Prevalence and correlates of emotional, physical, sexual, and financial abuse and potential neglect in the United States: the National Elder Mistreatment Study. Am. J. Public Health 100, 292–297. 10.2105/AJPH.2009.163089, PMID: 20019303 PMC2804623

[ref2] Alderson P. Clarke M. Mulrow C. D. Oxman A. D. (2008). Cochrane Handbook for Systematic Reviews of Interventions. Chichester, UK: John Wiley & Sons.

[ref3] Alt K. L. Nguyen A. L. Meurer L. N. (2011). The effectiveness of educational programs to improve recognition and reporting of elder abuse and neglect: a systematic review of the literature. J. Elder Abuse Negl. 23, 213–233. 10.1080/08946566.2011.584046, PMID: 27119527 PMC4852385

[ref4] Ayalon L. Lev S. Green O. Nevo U. (2016). A systematic review and meta-analysis of interventions designed to prevent or stop elder maltreatment. Age Ageing 45, 216–227. 10.1093/ageing/afv193, PMID: 26744361

[ref5] Baker P. R. A. Francis D. P. Hairi N. N. Othman S. Choo W. Y. (2016). Interventions for preventing abuse in the elderly. Cochrane Database Syst. Rev. 2016:CD010321. 10.1002/14651858.CD010321.pub2, PMID: 27528431 PMC7169376

[ref6] Cooper C. Barber J. Griffin M. Rapaport P. Livingston G. (2016). Effectiveness of START psychological intervention in reducing abuse by dementia family carers: randomized controlled trial. Int. Psychogeriatr. 28, 881–887. 10.1017/S1041610215002033, PMID: 26652193

[ref7] Cooper H. Hedges L. V. Valentine J. C. (2009). The Handbook of Research Synthesis and Meta-Analysis. Russell Sage Foundation.

[ref8] Davis R. C. Medina J. Avitabile N. (2001). Reducing Repeat Incidents of Elder Abuse: Results of a Randomized Experiment, Final Report. New York, NY: US Department of Justice.

[ref9] Dong X. Q. (2015). Elder abuse: systematic review and implications for practice. J. Am. Geriatr. Soc. 63, 1214–1238. 10.1111/jgs.13454, PMID: 26096395 PMC9958516

[ref10] Estebsari F. Dastoorpoor M. Mostafaei D. Khanjani N. Khalifehkandi Z. R. Foroushani A. R.. (2018). Design and implementation of an empowerment model to prevent elder abuse: a randomized controlled trial. Clin. Interv. Aging 13, 669–679. 10.2147/CIA.S158097 29713151 PMC5909776

[ref11] Fearing G. Sheppard C. L. McDonald L. Beaulieu M. Hitzig S. L. (2017). A systematic review on community-based interventions for elder abuse and neglect. J. Elder Abuse Negl. 29, 102–133. 10.1080/08946566.2017.1308286, PMID: 28339321

[ref12] Filinson R. (1993). An evaluation of a program of volunteer advocates for elder abuse victims. J. Elder Abuse Negl. 5, 77–94. 10.1300/J084v05n01_07

[ref13] Halpern S. H. Douglas M. J. (2005). Appendix: Jadad scale for reporting randomized controlled trials. Evidence-Based Obstetric Anesthesia, Oxford, UK: Blackwell Publishing Ltd., 237–238.

[ref14] Heck L. Gillespie G. L. (2013). Interprofessional program to provide emergency sheltering to abused elders. Adv. Emerg. Nurs. J. 35, 170–181. 10.1097/TME.0b013e31828ecc06, PMID: 23636048

[ref15] Hedges L. V. Tipton E. Johnson M. C. (2010). Robust variance estimation in meta-regression with dependent effect size estimates. Res. Synth. Methods 1, 39–65. 10.1002/jrsm.5, PMID: 26056092

[ref16] Hsieh H. F. Wang J. J. Yen M. F. Liu T. T. (2009). Educational support group in changing caregivers’ psychological elder abuse behavior toward caring for institutionalized elders. Adv. Health Sci. Educ. 14, 377–386. 10.1007/s10459-008-9122-6 18516696

[ref17] Khanlary Z. Maarefvand M. Biglarian A. Heravi-Karimooi M. (2016). The effect of a family-based intervention with a cognitive-behavioral approach on elder abuse. J. Elder Abuse Negl. 28, 114–126. 10.1080/08946566.2016.1141738, PMID: 26786905

[ref18] Livingston G. Barber J. Rapaport P. Knapp M. Griffin M. King D.. (2013). Clinical effectiveness of a manual based coping strategy programme (START, STrAtegies for RelaTives) in promoting the mental health of carers of family members with dementia: pragmatic randomised controlled trial. BMJ 347:f6276. 10.1136/bmj.f6276, PMID: 24162942 PMC3808082

[ref19] Marín-Martínez F. Sánchez-Meca J. (2010). Weighting by inverse variance or by sample size in random-effects meta-analysis. Educ. Psychol. Meas. 70, 56–73. 10.1177/0013164409344534

[ref21] Pellfolk T. J. E. Gustafson Y. Bucht G. Karlsson S. (2010). Effects of a restraint minimization program on staff knowledge, attitudes, and practice: a cluster randomized trial. J. Am. Geriatr. Soc. 58, 62–69. 10.1111/j.1532-5415.2009.02629.x, PMID: 20122041

[ref22] Ploeg J. Fear J. Hutchison B. Bolan G. (2009). A systematic review of interventions for elder abuse. J. Elder Abuse Negl. 21, 187–210. 10.1080/08946560902997181, PMID: 19827325

[ref23] Rabiei L. Mostafavi F. Masoudi R. Hassanzadeh A. (2013). The effect of family-based intervention on empowerment of the elders. J. Educ. Health Promot. 2:24. 10.4103/2277-9531.112700, PMID: 24083274 PMC3778562

[ref24] Rosen T. Elman A. Dion S. Delgado D. Demetres M. Breckman R.. (2019). Review of programs to combat elder mistreatment: focus on hospitals and level of resources needed. J. Am. Geriatr. Soc. 67, 1286–1294. 10.1111/jgs.15773, PMID: 30901078 PMC6561817

[ref25] Sacks D. Das D. Romanick R. Caron M. Morano C. Fahs M. C. (2012). The value of daily money management: an analysis of outcomes and costs. J. Evid. Based Soc. Work 9, 498–511. 10.1080/15433714.2011.581530, PMID: 23092378

[ref26] Tanner-Smith E. E. Tipton E. Polanin J. R. (2016). Handling complex meta-analytic data structures using robust variance estimates: a tutorial in R. J. Dev. Life-Course Cr. 2, 85–112. 10.1007/s40865-016-0026-5

[ref27] Teaster P. B. Nerenberg L. Stansbury K. L. (2003). A national look at elder abuse multidisciplinary teams. J. Elder Abuse Negl. 15, 91–107. 10.1300/J084v15n03_06

[ref28] Tipton E. (2015). Small sample adjustments for robust variance estimation with meta-regression. Psychol. Methods 20:375. 10.1037/met0000011, PMID: 24773356

[ref29] van Bavel M. Janssens K. Schakenraad W. Thurlings N. (2010). Elder Abuse in Europe. Background and Position Paper. 14:22.

[ref30] Vevea J. L. Woods C. M. (2005). Publication bias in research synthesis: sensitivity analysis using a priori weight functions. Psychol. Methods 10, 428–443. 10.1037/1082-989X.10.4.428, PMID: 16392998

[ref31] Viechtbauer W. (2010). Conducting meta-analyses in R with the metafor package. J. Stat. Softw. 36, 1–48. 10.18637/jss.v036.i03

[ref32] World Health Organization (2002). The Toronto declaration on the global prevention of elder abuse. Geneva: WHO. 3.

[ref34] Yon Y. Mikton C. R. Gassoumis Z. D. Wilber K. H. (2017). Elder abuse prevalence in community settings: a systematic review and meta-analysis. Lancet Glob. Health 5, e147–e156. 10.1016/S2214-109X(17)30006-2, PMID: 28104184

[ref35] Yon Y. Ramiro-Gonzalez M. Mikton C. R. Huber M. Sethi D. (2019). The prevalence of elder abuse in institutional settings: a systematic review and meta-analysis. Eur. J. Pub. Health 29, 58–67. 10.1093/eurpub/cky093, PMID: 29878101 PMC6359898

